# Asymmetric Clustering Index in a Case Study of 5-HT_1A_ Receptor Ligands

**DOI:** 10.1371/journal.pone.0102069

**Published:** 2014-07-14

**Authors:** Marek Śmieja, Dawid Warszycki, Jacek Tabor, Andrzej J. Bojarski

**Affiliations:** 1 Faculty of Mathematics and Computer Science, Jagiellonian University, Kraków, Poland; 2 Institute of Pharmacology, Polish Academy of Sciences, Kraków, Poland; UMIT, Austria

## Abstract

The automatic clustering of chemical compounds is an important branch of chemoinformatics. In this paper the Asymmetric Clustering Index (Aci) is proposed to assess how well an automatically created partition reflects the reference. The asymmetry allows for a distinction between the fixed reference and the numerically constructed partition. The introduced index is applied to evaluate the quality of hierarchical clustering procedures for 5-HT_1A_ receptor ligands. We find that the most appropriate combination of parameters for the hierarchical clustering of compounds with a determined activity for this biological target is the Klekota Roth fingerprint combined with the complete linkage function and the Buser similarity metric.

## Introduction

The rapidly growing number of compounds with a determined activity for a given molecular target leads to difficulties in using full, previously explored chemical spaces in virtual screening campaigns. Indeed, the use of a large number of ligands (e.g., the D_2_ receptor has 9180 different ligands in ChEMBL database v. 16 [1]) in predictive model development usually generates substantial computational costs. Moreover, for active compounds of any protein target, large groups of similar ligands may significantly disrupt the search results, limiting virtual hits to close analogs of over-representative input structures [2], [3]. As a consequence, an appropriate clustering of the ligands' chemical space is of primary importance [4].

Manual (knowledge-based) clustering is usually the first choice for small groups of ligands because it provides the most natural partitions. However, for more abundant sets, this approach is time-consuming and requires extensive chemical knowledge (e.g., the manual clustering of 3616 5-HT_1A_ receptor ligands performed by Warszycki et al. [5] took a couple of weeks). Therefore, automatic clustering algorithms are frequently used for categorizing chemical compounds. Consequently, it is crucial to employ indices that can verify how similar a numerically constructed partition is to the reference created by experts.

Unlike experts, who intuitively recognize and classify chemical structure, automatic clustering algorithms require molecule to be translated into an appropriate form. This is usually achieved by application of fingerprints which transform chemical structure on a bitstring, where “1” and “0” correspond to a presence or absence of a particular chemical pattern, respectively [6], [7]. Next, fingerprints can be compared using a similarity metric evaluating how much the compounds are similar [8]. Moreover, hierarchical clustering procedures require, the linkage function which determines the “distance” between two groups of compounds. Since there are a lot of available fingerprints, metrics and linkage functions, the number of their combinations is indeed quite high, which makes finding the most appropriate one, for a particular task, relatively difficult.

Several methods have been proposed to compare clusterings [9]. The most popular techniques are based on counting pairs of elements classified in the same way in both partitions, such as the rand index [10] and its modifications [11], [12]. Another group of methods uses normalized mutual information to quantify the information shared by the clusterings [13], [14]. An interesting approach for comparing partitions relies on measuring the distance between clusterings with the use of information theory [15]. The main feature of these indices is their symmetry, which makes them suitable for finding the similarities between clusterings.

In the present study, we introduce the Asymmetric Clustering Index (Aci) for comparing two partitions. The asymmetry allows the index to distinguish between the fixed reference (which by default, denotes the expert manual partition) 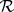 and the numerically constructed partition 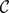. As a consequence, the Aci is capable of measuring how well a given partition reflects the reference (not conversely). This index is defined as the ratio of the mutual information 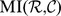 to the entropy 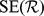: 
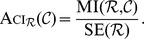



The Aci is reminiscent of the indices proposed in [13], [14] but, due to its different normalization factor, has an asymmetry feature.

**Figure 1:**
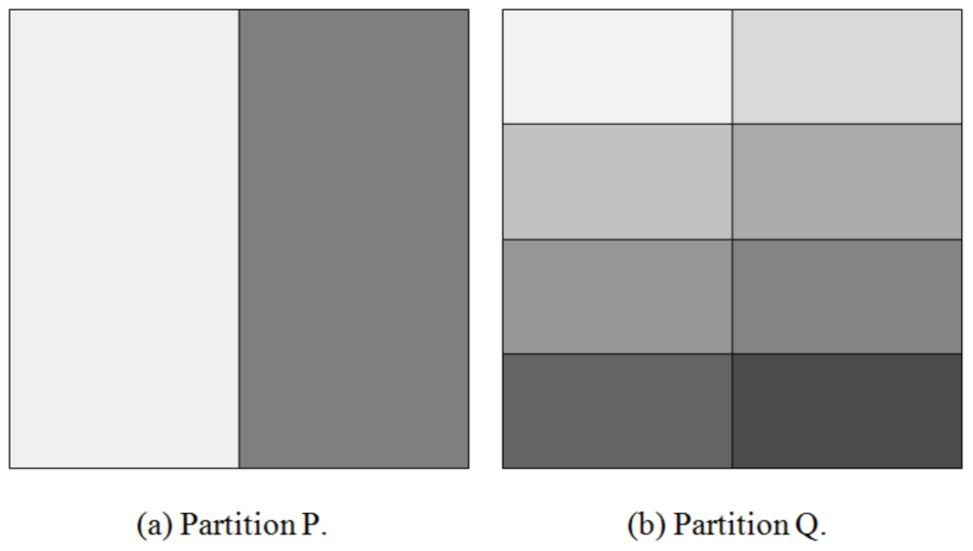
Presentation of the Aci. Partition 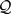 contains more information than partition 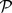; thus, 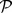 can be restored from 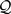 by merging four pairs of sets. In particular, 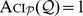 and 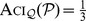.

The basic properties of the Aci are presented in Figure 1 and are listed below:

- it takes on values between 0 and 1,
- the reference can be recovered from the partition by merging selected groups if 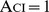,
- for the partitions that do not share any information, 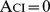.

**Figure 2:**
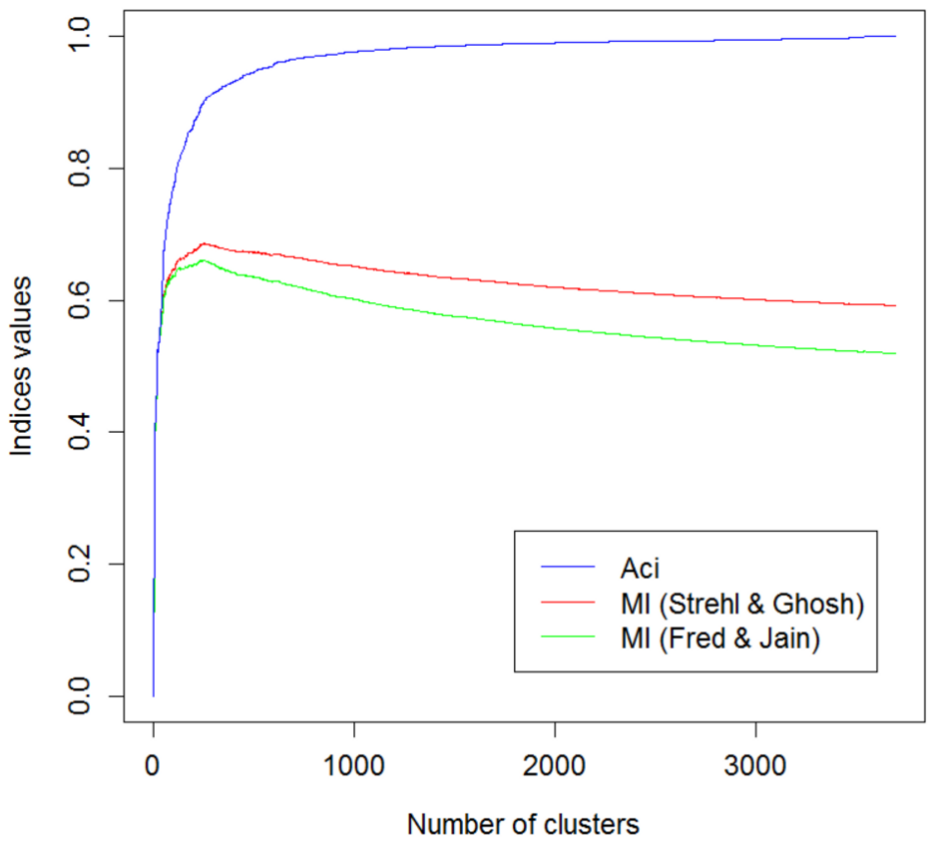
Comparison between the Aci and symmetric indices based on mutual information. These indices were evaluated based on the reference reported by Warszycki et al. [5], and the partitions were obtained from hierarchical clusterings performed with the Klekota Roth fingerprint combined with the Buser similarity metric and the complete linkage function.

Therefore, for successively subdivided partitions, the Aci converges to 1, in contrast to symmetric indices. Figure 2 presents the values of the Aci and other two similarity indices based on mutual information for a conducted experiment. When the number of clusters obtained in the hierarchical clustering is greater, the reference is better reflected by the partition. As a result, the Aci takes gradually higher values in contrast to the other indices. This behavior allows for a straightforward interpretation of the Aci – values close to 1 indicate that the numerically constructed partition contains much information about the reference.

To determine the optimal conditions reaching the maximum Aci values, 8 fingerprint types, 4 similarity metrics and 4 linkage functions were applied to a hierarchical clustering of the full chemical space of 5-HT_1A_ receptor ligands. As a reference, the manually constructed partition of Warszycki et al. [5] was taken, which generally follows the classification of 5-HT_1A_ R described in the literature [16], [17]. The best clustering was achieved for a combination of the Klekota Roth fingerprint, the Buser similarity metric and the complete linkage function, which was then verified in an additional clustering experiment on a collection of compounds belonging to two explicitly different chemical classes. Thus, in further studies, automatic clustering should be performed with these parameters.

## Materials and Methods

The Aci measures how well the automatically performed partition 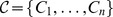 reflects the reference 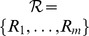. This index is obtained by normalizing the mutual information 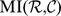 by the entropy 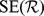: 
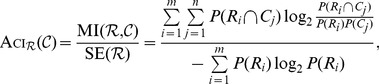
(1)where 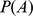 denotes the probability that an element belongs to set 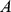. The above metric quantifies the percent of information that 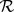 delivers about 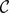.

**Figure 3:**
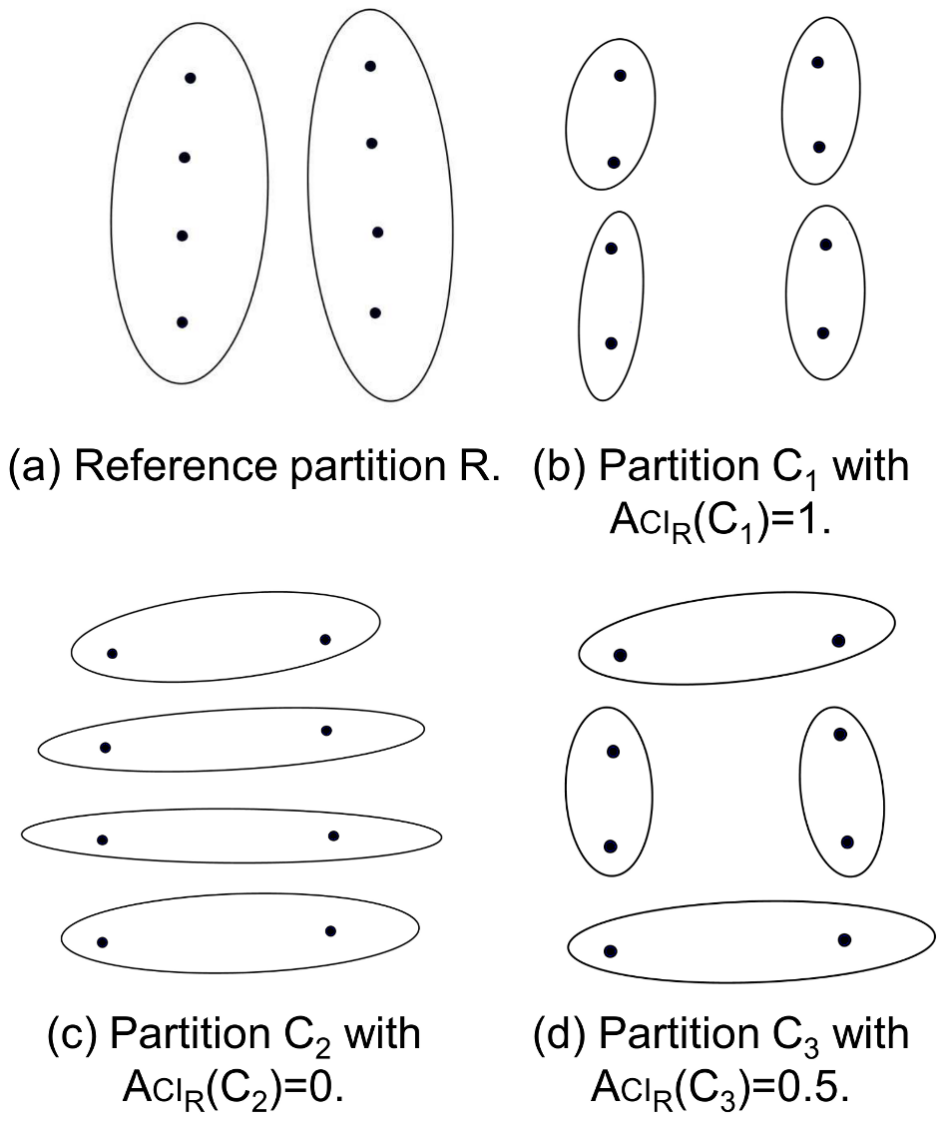
Illustration of the Aci. Partition 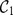 fully reflects the reference, 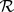 (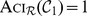). In contrast, partition 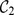 is random with respect to the reference – the two results do not share any information (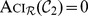). Partition 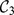 is a combination of the two previous situations – half of the reference can be recovered from this clustering (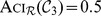).

The Aci attains a maximal value of 1 if the reference and the numerically constructed partitions are identical. However, as shown in Figure 3, we also obtain 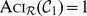 when the reference is subdivided into smaller clusters; clearly, this automatically constructed clustering contains at least as much information as the reference. Consequently, the reference can be reconstructed from the numerically obtained partition by merging selected groups. In contrast, if the partition 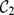 is random with respect to 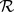, then the clusterings are completely different, which results in 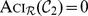. This case holds, for example, when every cluster of 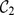 contains an equal number of elements in comparison to each cluster of 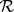. One can also consider a composition of these two examples.

In the case of hierarchical clustering, for every two partitions obtained by cutting at different levels, one partition is a subdivision of the second. Furthermore, when a partition has as many groups as the number of data-set elements (every cluster is a one-element set), then it contains information about every possible partition. Clearly, for a high number of clusters, practically all information about the reference partition can be deduced from the partition numerically constructed by an arbitrary clustering algorithm. In contrast, a partition cannot fully reflect the reference if it has fewer elements. Consequently, one of the possible methods for determining the optimal number of clusters is to maximize a selected measure of dispersion, e.g., the standard deviation or entropy. In other words, a given number of clusters is optimal for the Aci if it maximally distinguishes among the partitions (with respect to the corresponding Aci values). Numerical examples indicate that reasonable results are obtained when approximately twice the number of groups are taken in comparison to the reference division (see the next section for more details).

The idea of the Aci is based on information theory; in particular, this index involves the notions of entropy and mutual information content. The Shannon entropy, introduced as a measure of channel capacity in digital communications [18], is also used to quantify the information contained in the clustering [19]. Formally, the Shannon entropy (SE) of an 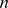-element partition 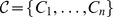 is defined by 
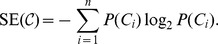



In the case of a one-element partition, the cluster of each element is known; therefore the SE equals 0. In contrast, if no information about the position of any element is provided (every cluster is equally probable), then the SE attains a maximum.

To compare two clusterings, the basic idea of the SE needs to be extended by defining the mutual information (MI). The MI determines the amount of information shared between partitions and is defined by [20]

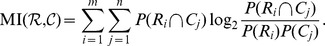



**Figure 4:**
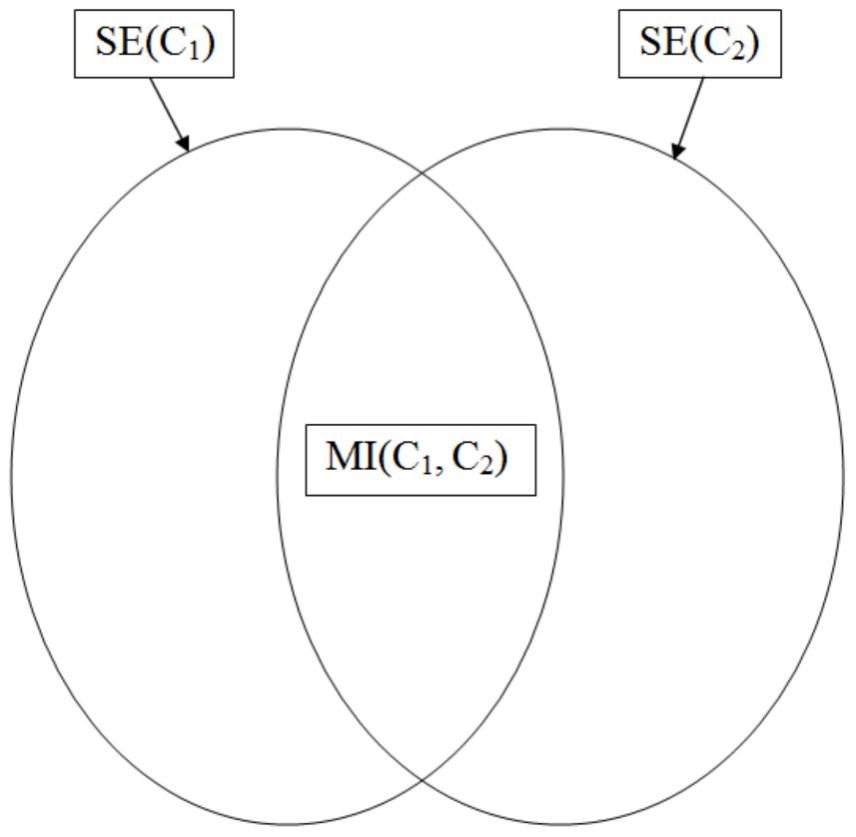
Comparison between entropy and mutual information. Each region describes the information provided by a particular clustering [20].

The relations between the introduced quantities are presented in Figure 4.

It is straightforward to demonstrate that the mutual information is symmetric [20], i.e., 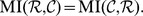



As mentioned in the Introduction, this property allows only one to evaluate the similarity between partitions. To define an asymmetric index that measures how well the reference can be recovered from the numerically created partition, the normalization by the entropy of reference partition is used, giving the following formula: 
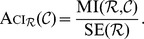



By [20], we have 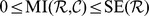, which leads to: 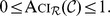



## Results

One of the most popular techniques used to divide chemical compounds is hierarchical clustering [21]. The strength of this approach lies in the deterministic nature of the algorithm and the constructed hierarchical structure of clusters. This method requires the specification of several input parameters, but there is no unified methodology for determining which parameters will provide the best results. The Aci will be applied to determine the combination of parameters that best reflect the reference partition of 5-HT_1A_ receptor ligands.

As a reference, the manually constructed partition of Warszycki [5] was utilized. All ligands (retrieved from approximately 520 published papers) used for this clustering were extracted from ChEMBL database version 5 (August 2010) [1]. Ligands with an inhibition constant (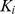) of less than or equal to 100 nM were considered active; only these ligands were used for this clustering study.

**Figure 5:**
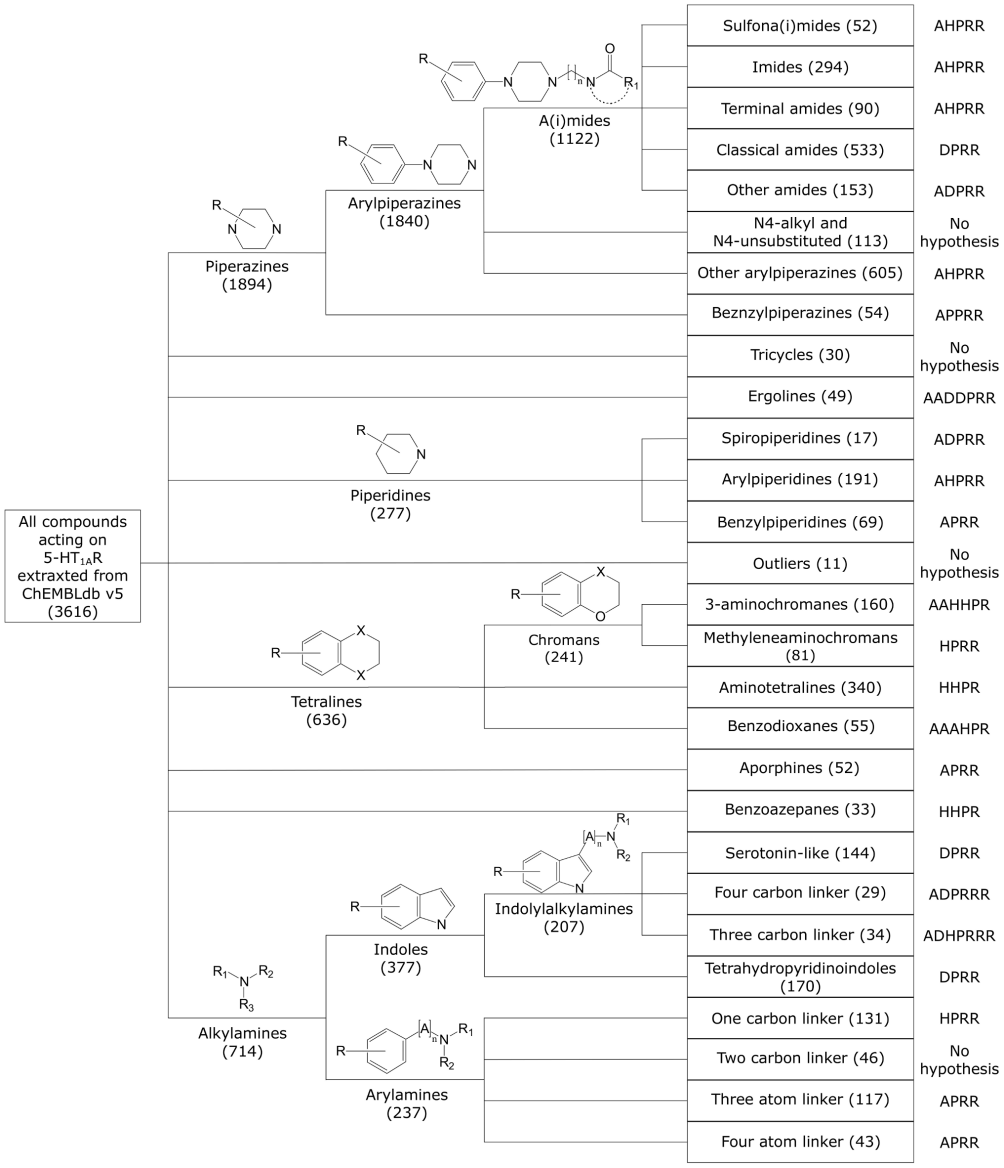
The results obtained by manual clustering of 5-HT_1A_ receptor ligands. This process is described in Warszycki et al. [5].

The manual clustering generally follows the classification of 5-HT_1A_ ligands described in the literature (9 basic classes) [16], [22], [23]; however, some additional subgroups were then created, e.g., for arylpiperazines [17]. In the case of alkylamines (714 compounds), indole derivatives were first extracted and, with the exception of the tetrahydropyridoindoles, were divided depending on the distance between two crucial pharmacophore features: an aromatic system and a basic nitrogen atom. The entire procedure resulted in 28 clusters, each containing 17 to 605 compounds [5] (see Figure 5).

In this study, three types of hierarchical clustering parameters were examined. The study focused on determining the optimal Aci values from a combination of eight fingerprint representations (Table 1), four linkage functions (Table 2) and four similarity metrics (Table 3). Both recently published works [8], [24] and our experience, supported by preliminary studies, indicate that these four metrics are the most relevant for clustering purposes.

**Table 1:** The characteristics of fingerprints, with the abbreviations used in this work.

Fingerprint	Abbreviation	Length of fingerprint
EState fingerprint [25]	estate	79
Fingerprint [26]	fingerprint	1024
Extended fingerprint [27]	extended	1024
Graph only fingerprint [27]	graph only	1024
Klekota Roth fingerprint [28]	KRFP	4860
MACCS fingerprint [29]	maccs	166
PubChem fingerprint [27]	pubchem	881
Substructure fingerprint [27]	substructure	308

All fingerprints were generated in PaDEL software [27].

**Table 2:** Linkage functions for two sets [30].

Name	Formula
Average	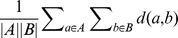
Centroid	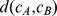
Complete	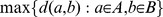
Single	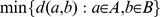

Used marks in the formula: 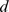 – metric, 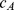 – center of set 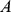, 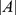 – cardinality of set 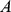.

**Table 3:** Similarity metrics [8].

Name	Formula
Buser	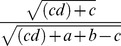
Dice	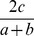
Tanimoto	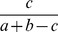
Yule	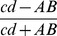

Used marks in the formula: 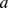 – on bits in structure 1, 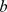 – on bits in structure 2, 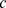 – on bits in both 1 and 2, 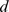 – off bits in both 1 and 2, 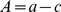, 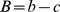.

**Figure 6:**
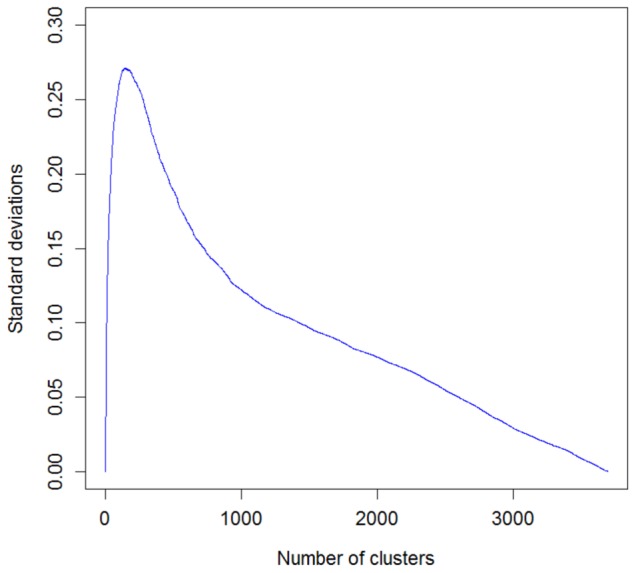
Standard deviations of Aci values collected for the 128 combinations of hierarchical clustering parameters.

**Figure 7:**
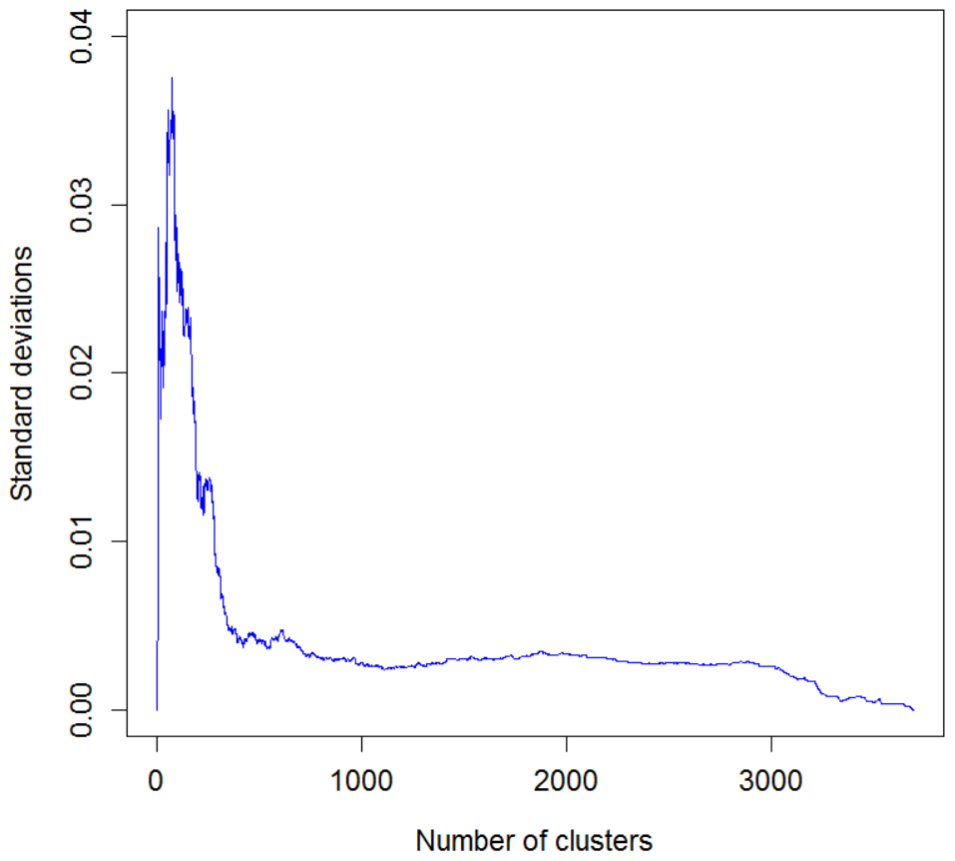
Standard deviations of Aci values collected for the 12 best combinations of hierarchical clustering parameters. These combinations correspond to the highest mean Aci values over all possible cluster numbers. The maximum occurs for the cluster numbers between 50 and 80.

To determine the optimal number of clusters for the Aci, an additional experiment was conducted. The Aci was evaluated for all combinations of linkage functions, fingerprint representations and similarity metrics (total of 128 cases). The corresponding standard deviations for each number of clusters were calculated, as shown in Figure 6. Because this study focuses on selecting the optimal parameters, standard deviations were also computed for 12 combinations that provided the highest mean Aci values (averaged over all possible numbers of groups). This restriction reduced the number of clusters for which the maximal discrimination was attained (Figure 7). As a consequence, a total of 50 groups was chosen as a reasonable compromise between accuracy and complexity for this model.

The results (Table 4) shows that the choice of linkage function has the most significant impact on the clustering results, regardless of the fingerprint representation or similarity metric (clearly, this holds only for the types of metrics employed herein). The mean Aci values calculated for the clusterings for particular linkage functions indicate that optimal performance is obtained with the complete linkage function.

**Table 4:** Complete linkage function rankings.

Linkage function	Aci
Complete	0.51
Average	0.40
Centroid	0.09
Single	0.04

Mean Aci values obtained for fixed four types of linkage functions and various types of fingerprints and similarity metrics.

**Figure 8:**
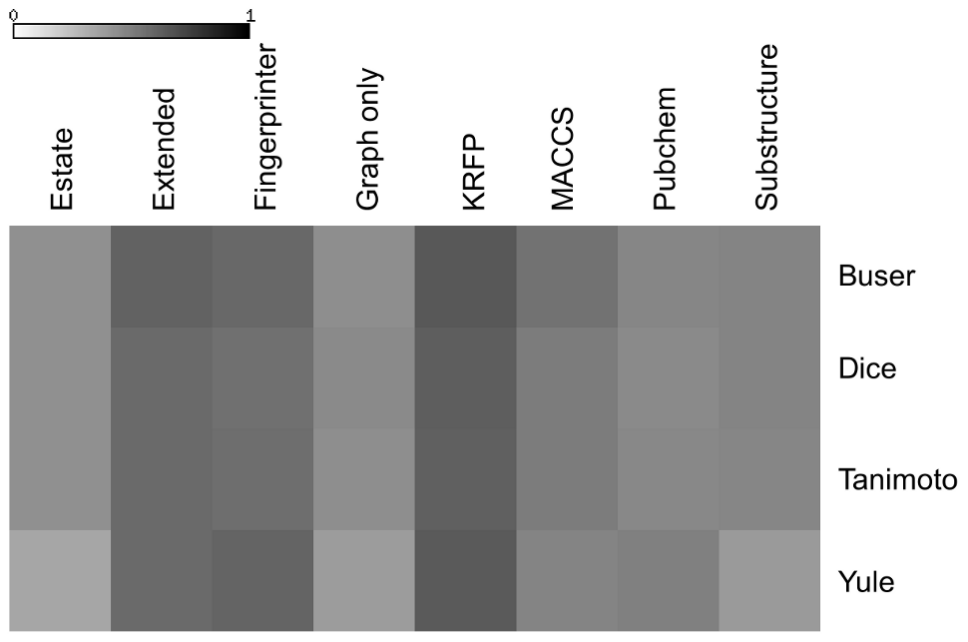
Aci values for hierarchical clusterings with the complete linkage function.

**Figure 9:**
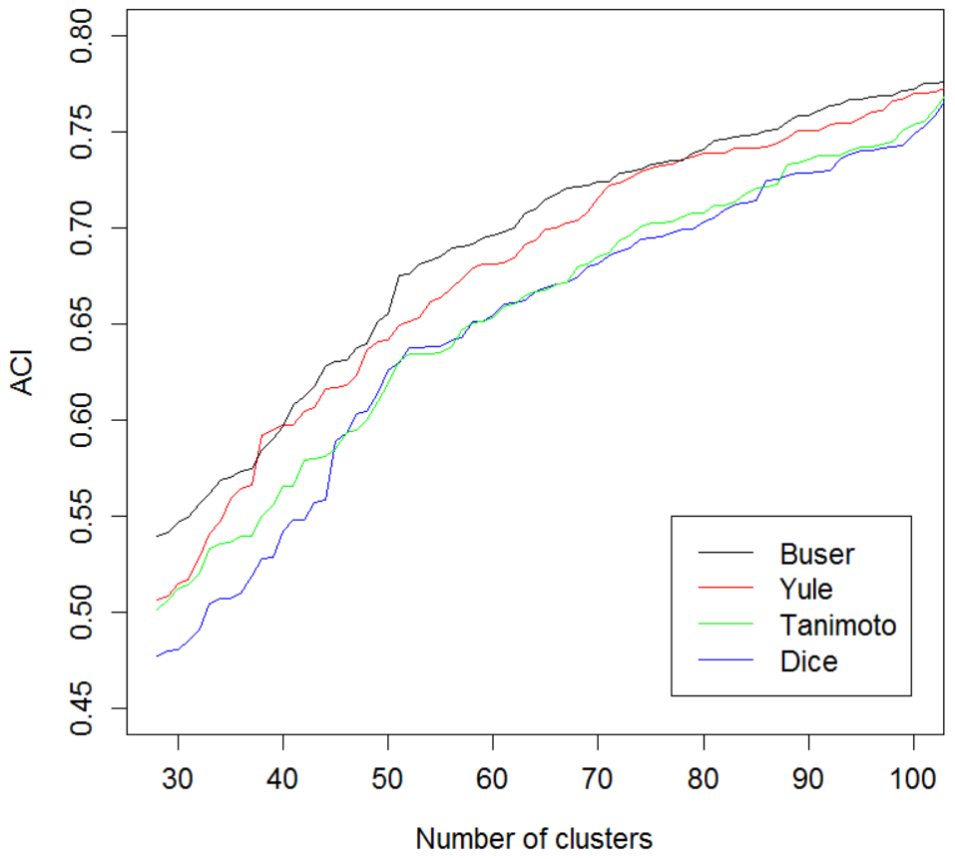
Aci values for hierarchical clusterings. The number of groups ranged from 28 to 100. Results are presented for the complete linkage function, the Klekota Roth fingerprint and four different similarity metrics.

An analysis of the Aci values for partitions with the complete linkage function and various fingerprint representations and similarity metrics (Figure 8) points out the superiority of the KRFP fingerprint for all four metrics. The impact of the similarity metrics was then assessed by varying the number of clusters from 28 to 100 in series of experiments with the complete linkage function and the KRFP molecular representation. This investigation (Figure 9) demonstrated the superiority of the Buser similarity metric over the remaining three types for almost all cluster numbers.

Next, the ability of the optimally designed hierarchical clustering to separate compounds belonging to different chemical classes was additionally evaluated. For this purpose, three partitioning experiments were performed: the separation of (a) arylpiperazines with a sulfona(i)mide fragment from aporphines, (b) benzodioxans from benzylpiperazines and (c) N4-alkyl and N4-unsubstituted arylpiperazines from arylalkilamines with a three-atom linker. In the first two cases, the automatic process perfectly or very closely (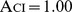 and 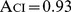, respectively) reflected the reference clustering. In the third case the obtained result was highly unsatisfactory (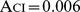); however, increasing the number of clusters up to three significantly improve the quality of the separation (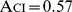). Fixing the number of clusters to 6 resulted in 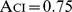, while 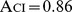 was obtained for eight clusters. These results confirm the need to enforce a greater number of groups in the clustering process than expected.

In conclusion, the experiments demonstrate that the automatic hierarchical clustering of 5-HT_1A_ receptor ligands provides the best results when implemented with the complete linkage function, the KRFP fingerprint representation and the Buser similarity metric. It is worth mentioning that satisfactory results are also obtained with the use of three other metrics – the Tanimoto, Yule and Dice metrics.

## Conclusion

This paper introduces a straightforward asymmetric index, the Aci, which allows one to evaluate how well a numerically constructed partition reflects the reference. The highest Aci was consistently obtained for hierarchical clustering based on the complete linkage function, the Klekota-Roth fingerprint and the Buser similarity metric, suggesting the application of these parameters for other groups of biologically active compounds. This approach was verified using a manually constructed partition of active 5-HT_1A_ ligands [5].

An SDF file containing the full collection of 3616 compounds is available free of charge via the Internet at http://skandal.if-pan.krakow.pl/5-HT1A_ligands.sdf. To obtain a hierarchical clustering of the considered chemical space, the hclust function of R software was used. A sample R code used for the Aci calculation is available free of charge at http://skandal.if-pan.krakow.pl/aci.R.
